# Supplementary figures and images for: An interdisciplinary study around the reliquary of the late cardinal Jacques de Vitry
Source: PLoS One. 2019 Feb 22;14(2):e0201424. doi: 10.1371/journal.pone.0201424 (PMC6386372; doi:10.1371/journal.pone.0201424)

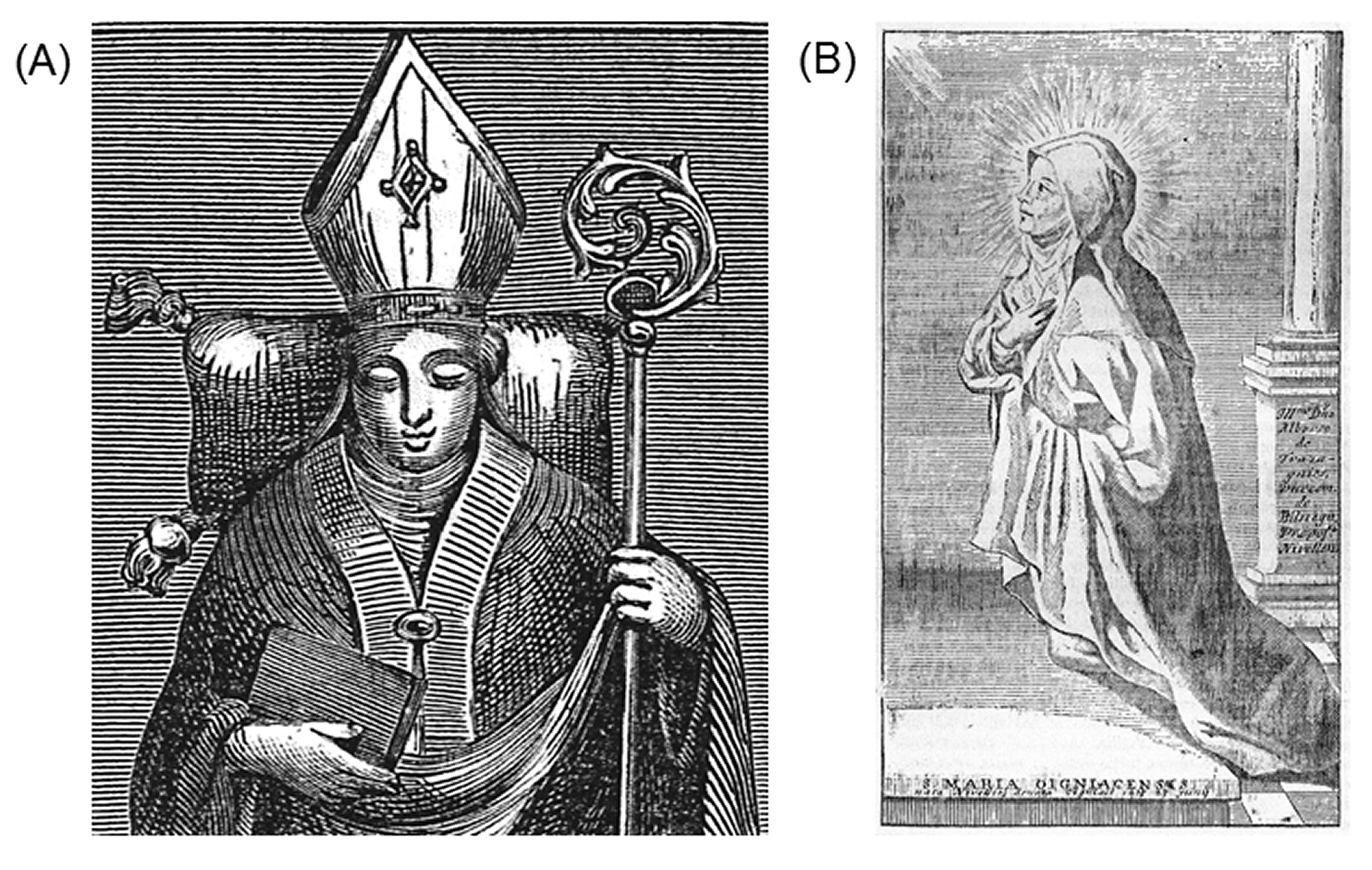

Supplement: S1 Fig — (A) Cardinal Jacques de Vitry on his deathbed (A. Marminia and E. Borne, engraving). (B) Saint Marie d’Oignies (engraving). Republished from “De B. Maria Oigniacensi in Namurcensi Belgii dioecesi. Appendix” in “Acta Sanctorum” under a CC BY license, with permission from Société des Bollandistes, original copyright 1867. (TIF) [file pone.0201424.s001.tif]

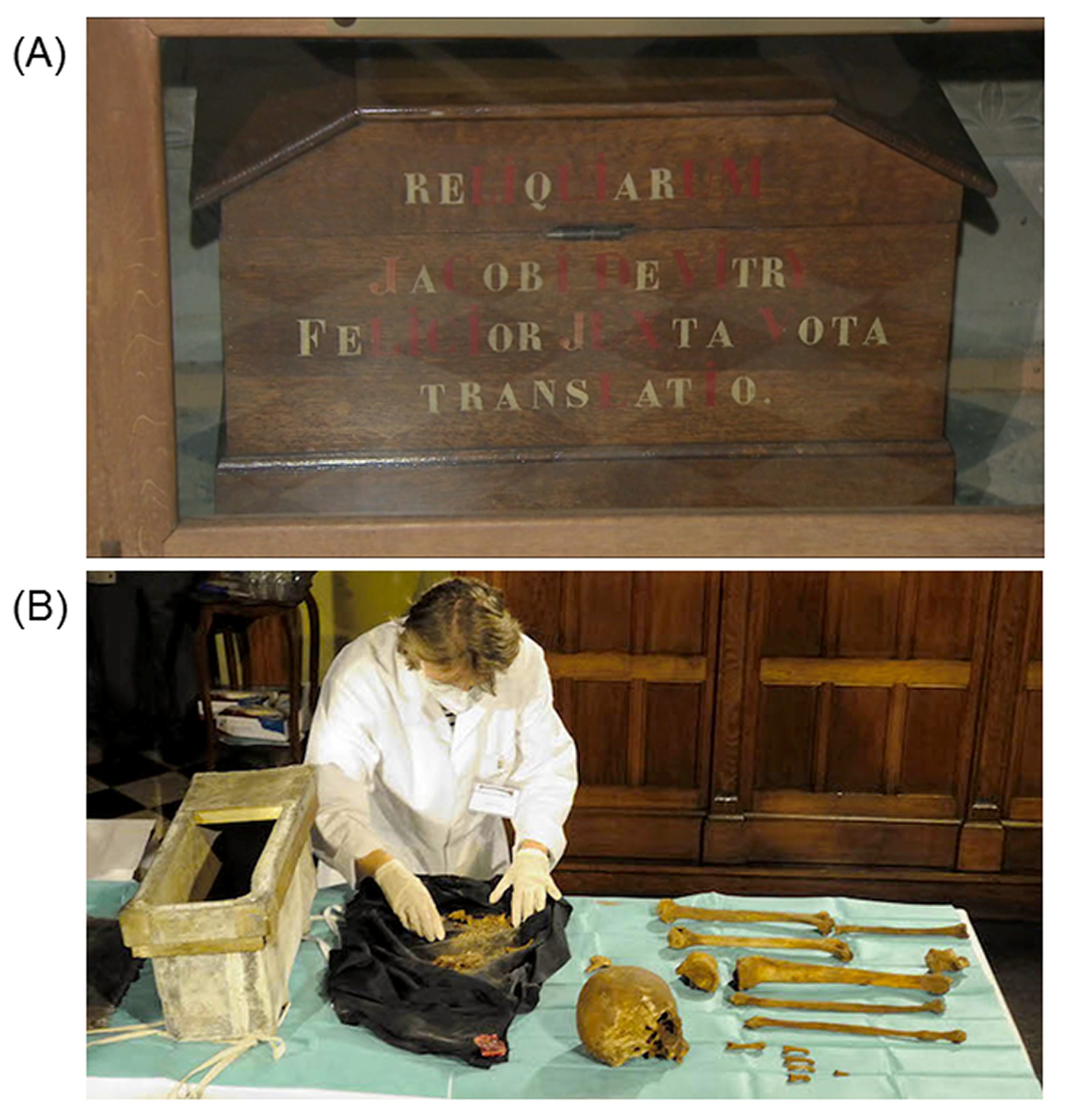

Supplement: S2 Fig — (A) The reliquary. (B) The remains found in the reliquary after opening on 8th September 2015. Reprinted under a CC BY license with permission from Vedrin, Guy Focant, original copyright 2015. (TIF) [file pone.0201424.s002.tif]

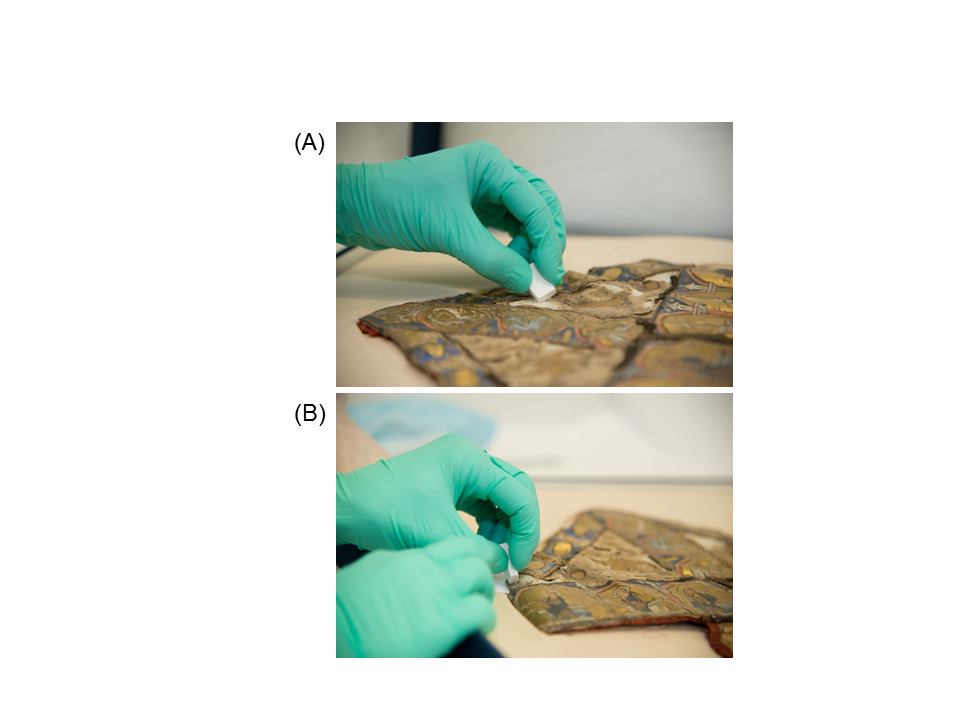

Supplement: S3 Fig — Gentle rubbing of the parchment surface with a PVC eraser for proteomic analyses. Reprinted under a CC BY license with permission from Vedrin, Guy Focant, original copyright 2012. (TIF) [file pone.0201424.s003.tif]
