# Supplementary material for: An interdisciplinary study around the reliquary of the late cardinal Jacques de Vitry
Source: PLoS One. 2019 Feb 22;14(2):e0201424. doi: 10.1371/journal.pone.0201424 (PMC6386372; doi:10.1371/journal.pone.0201424)
Supplement: S1 Table — (DOCX) [file pone.0201424.s004.docx]

**S1 Table. Isotopic fractionations of carbon (δ^13^C) and nitrogen (δ^15^N) according to diet.**

| **Bone collagen from animals having a 100% diet of** | **δ^13^C (‰)** | **δ^15^N (‰)** |
| --- | --- | --- |
| C3-plants | -21 | +5 |
| Meat C3-herbivores | -18 | +8 |
| C-4 plants | -7 | +5 |
| Marine food | -13 | +18 |
| River fish | -24 | +16 |
| Lake fish | -20 | +16 |

Data reproduced from Lanting and van der Plicht (1996, 1998).
